# Male reproductive strategy explains spatiotemporal segregation in brown bears

**DOI:** 10.1111/1365-2656.12055

**Published:** 2013-03-05

**Authors:** Sam MJG Steyaert, Jonas Kindberg, Jon E Swenson, Andreas Zedrosser

**Affiliations:** 1 Institute of Wildlife Biology and Game Management, University of Natural Resources and Life SciencesVienna, A-1180, Austria; 2 Department of Ecology and Natural Resource Management, Norwegian University of Life SciencesÅs, NO-1432, Norway; 3 Department of Wildlife, Fish and Environmental Studies, Swedish University of Agricultural SciencesUmeå, SE-90183, Sweden; 4 Norwegian Institute for Nature ResearchTrondheim, NO-7485, Norway; 5 Faculty of Arts and Sciences, Department of Environmental and Health Studies, Telemark University CollegeBø, NO-3800, Norway

**Keywords:** nonparental infanticide, reproductive strategy, resource selection, risk effects, risk factor, segregation, sexual selection, sexual size dimorphism, *Ursus arctos*

## Abstract

**1.** Spatiotemporal segregation is often explained by the risk for offspring predation or by differences in physiology, predation risk vulnerability or competitive abilities related to size dimorphism.

**2.** Most large carnivores are size dimorphic and offspring predation is often intraspecific and related to nonparental infanticide (NPI). NPI can be a foraging strategy, a strategy to reduce competition, or a male reproductive strategy. Spatiotemporal segregation is widespread among large carnivores, but its nature remains poorly understood.

**3.** We evaluated three hypotheses to explain spatiotemporal segregation in the brown bear, a size-dimorphic large carnivore in which NPI is common; the ‘NPI – foraging/competition hypothesis', i.e. NPI as a foraging strategy or a strategy to reduce competition, the ‘NPI – sexual selection hypothesis’, i.e. infanticide as a male reproductive strategy and the ‘body size hypothesis’, i.e. body-size-related differences in physiology, predation risk vulnerability or competitive ability causes spatiotemporal segregation. To test these hypotheses, we quantified spatiotemporal segregation among adult males, lone adult females and females with cubs-of-the-year, based on GPS-relocation data (2006–2010) and resource selection functions in a Scandinavian population.

**4.** We found that spatiotemporal segregation was strongest between females with cubs-of-the-year and adult males during the mating season. During the mating season, females with cubs-of-the-year selected their resources, in contrast to adult males, in less rugged landscapes in relative close proximity to certain human-related variables, and in more open habitat types. After the mating season, females with cubs-of-the-year markedly shifted their resource selection towards a pattern more similar to that of their conspecifics. No strong spatiotemporal segregation was apparent between females with cubs-of-the-year and conspecifics during the mating and the postmating season.

**5.** The ‘NPI – sexual selection hypothesis’ best explained spatiotemporal segregation in our study system. We suggest that females with cubs-of-the-year alter their resource selection to avoid infanticidal males. In species exhibiting NPI as a male reproductive strategy, female avoidance of infanticidal males is probably more common than observed or reported, and may come with a fitness cost if females trade safety for optimal resources.

## Introduction

Predation is a strong selective force that can directly affect an individual's fitness through its or its offspring's death (Lima & Bednekoff 1998). Predation can also indirectly affect an individual's fitness due to costs associated with antipredator behaviour and predation risk (Brown & Kotler 2004; Creel & Christianson 2008), for example by affecting foraging decisions, habitat choice and activity budgets, which can be sex and age specific (Lima & Bednekoff 1998; Brown, Kotler & Bouskila 2001). Animals can respond rapidly to changing predation risk regimes (Lima & Bednekoff 1998; Brown & Kotler 2004), and potential risk factors can be identified by relating characteristics (e.g. temporal, sex specific) of each risk factor with temporal variation in animal behaviour (Lima & Bednekoff 1998; Brown & Kotler 2004).

In large carnivores, adult survival should be little influenced by predation. Female reproductive success is, however, often affected by nonparental conspecific killing of dependent young (nonparental infanticide, NPI) (Swenson 2003; Rode, Farley & Robbins 2006). Hrdy (1979) recognized three adaptive forms of NPI. First, NPI can be a foraging strategy, in which unrelated dependent young are exploited as food items by individuals of both sexes that are large enough to subdue the victim. The size and vulnerability of the infant are more important than its age (Hrdy & Hausfater 1984). Secondly, NPI can be a strategy to reduce competition for the perpetrator and its kin. The predictions for NPI as a competitive strategy are the same as for NPI as a foraging strategy, i.e. perpetrators of both sexes kill unrelated dependent offspring, and the vulnerability of the victim is more important than its age (Hrdy & Hausfater 1984). Thirdly, NPI can be an adaptive male reproductive strategy, when males gain mating opportunities by killing unrelated dependent young [i.e. sexually selected infanticide (SSI)] (Hrdy 1979). SSI shortens the time to the victimized female's next oestrus and a perpetrating male may sire her next litter (Hrdy 1979). SSI is common in size-dimorphic species with a polygamous mating system (van Schaik 2000) and occurs during the mating season in seasonal breeders that have extended maternal care and lactational anoestrous (Zedrosser *et al*. 2009). Nonadaptive forms of NPI, including social pathology or accidental infant killing, receive little support in the literature (Hrdy 1979; van Schaik & Janson 2000).

NPI is an important selective pressure in the evolution of mammalian mating systems (Wolff & Macdonald 2004), and female adaptive behaviour that minimizes infanticide probably led to the evolution of infanticide counterstrategies (Ebensperger 1998). Such strategies include pregnancy termination, aggression, group defence, multi-male mating, territoriality and avoidance of potentially infanticidal conspecifics (Ebensperger 1998). Avoiding potentially infanticidal conspecifics can lead to spatiotemporal segregation among individuals of certain sex, age or reproductive status (Wielgus & Bunnell 1994; Loseto *et al*. 2006; Libal *et al*. 2011). Spatiotemporal avoidance of infanticidal individuals is an obvious counterstrategy, but conclusive evidence for it is rare and mainly involves group-living species (Ebensperger & Blumstein 2007) such as lions (*Panthera leo*) (Pusey & Packer 1994) and Hanuman langurs (*Presbytis entellus*) (Hrdy 1979).

Because sexual size dimorphism is common among species that exhibit NPI (van Schaik 2000), segregation among sex and age classes can also arise because of size-related differences in physiology, predation risk vulnerability or competitive abilities (Ruckstuhl 2007; Main 2008). Conclusive evidence for sexual segregation is common for group-living ungulate species, such as sheep (*Ovis canadensis, O. ammon*) and red deer (*Cervus elaphus*) (Ruckstuhl 2007; Main 2008; Singh *et al*. 2010).

The brown bear (*Ursus arctos*) is a solitary, size-dimorphic carnivore with a polygamous mating system (Steyaert *et al*. 2012a). Mortality in cubs-of-the-year (<1-year-old; hereafter termed ‘cubs’) varies from 4% to 66% among populations (Miller 1990; Sæther *et al*. 1998), and NPI is considered as the major cause of death (Bellemain, Swenson & Taberlet 2006; Garshelis 2009; Steyaert *et al*. 2012a). NPI is committed by both sexes (Hessing & Aumiller 1994; Miller, Sellers & Keay 2003; Ben-David, Titus & Beier 2004), albeit mostly by males (McLellan 1994; Craighead, Sumner & Mitchell 1995; Swenson *et al*. 1997). Evidence for the three functional types of NPI appears to vary across populations. For example NPI as a foraging strategy and as strategy to reduce competition has been suggested for several North American populations (Hessing & Aumiller 1994; Craighead, Sumner & Mitchell 1995; Miller, Sellers & Keay 2003; McLellan 2005), whereas SSI probably explains NPI in some North American and Scandinavian populations (Wielgus & Bunnell 1995; Swenson *et al*. 1997; Bellemain, Swenson & Taberlet 2006; Libal *et al*. 2011). Female counterstrategies to NPI in brown bears include direct defence (Craighead, Sumner & Mitchell 1995), promiscuity and multiple paternity (Bellemain, Swenson & Taberlet 2006), selecting escape habitat (Pearson 1975; Swenson 2003), elusiveness (Dahle & Swenson 2003) and avoidance of sites with high infanticide risk (e.g. clumped food resources) (Wielgus & Bunnell 1995; Ben-David, Titus & Beier 2004; Rode, Farley & Robbins 2006).

Brown bear populations throughout their geographical range have a spatiotemporally structured social organization (often termed ‘segregation’ or ‘despotism’) (Wielgus & Bunnell 1994; Craighead, Sumner & Mitchell 1995; Ben-David, Titus & Beier 2004; Rode, Farley & Robbins 2006; Libal *et al*. 2011). The ultimate and proximate causes of spatiotemporal structure in relation to sex, age and reproduction (hereafter termed ‘spatiotemporal segregation’) are often poorly understood and are a topic of debate (Miller, Sellers & Keay 2003; McLellan 2005).

Our objective was to explain spatiotemporal segregation in a nonsocial carnivore, the brown bear, in an environment where food sources are relatively evenly distributed across a human-influenced landscape. We quantified spatiotemporal segregation based on resource selection functions and maps (diurnally and seasonally), and evaluated three hypothesis that may explain spatiotemporal segregation among three reproductive classes of bears: adult males (≥5 years), lone adult females (≥5 years, hereafter termed ‘lone females’) and females with cubs-of-the-year (hereafter termed ‘females/cubs’).

Because the predictions for explaining NPI as a foraging strategy and as a strategy to reduce competition are the same (Hrdy & Hausfater 1984), we formulate hypothesis 1 (H1) as the ‘NPI – foraging/competition hypothesis’ to explain spatiotemporal segregation in our study population. H1 predicts that (a) spatiotemporal segregation is absent between lone females and adult males throughout the year; (b) that females/cubs strongly segregate from adult males throughout the year; and (c) that females/cubs segregate from lone females throughout the year, albeit less strong compared with adult males. We formulate hypothesis 2 (H2) as the ‘NPI – sexual selection hypothesis’, which postulates that infanticide as a male reproductive strategy causes spatiotemporal segregation. H2 predicts (a) no segregation between adult males and lone females throughout the year; (b) strong segregation between females/cubs and adult males during the mating season, but not during the postmating season and (c) segregation between females/cubs and lone females, but only during the mating season, when adult males and lone females often consort. The ‘body size hypothesis’ (H3) postulates that body size-related differences in physiology, predation risk vulnerability or competitive ability cause spatiotemporal segregation. H3 predicts that (a) adult males and lone females do not segregate during the mating season, but do so during the postmating season; (b) adult males and females/cubs segregate throughout the year and (c) females/cubs and lone females segregate during the mating season (when males and lone females consort for mating), but not during the postmating season.

## Materials and methods

The study was conducted in an intensively managed boreal forest in south-central Sweden (∼61°N, 15°E), with a dense network of logging roads (0·7 km per km^2^) and few high-traffic roads (0·14 km per km^2^) (Martin *et al*. 2010). The human population density is low, with few settlements and isolated houses (mainly holiday cabins) (Martin *et al*. 2010). Human presence is most pronounced during summer and fall, and mainly related to hunting and berry picking (Ordiz *et al*. 2011). Brown bear population density is about 30 individuals per 1000 km^2^ and the population is intensively hunted (from 21 August until 15 October) (Bischof *et al*. 2009). Average asymptotic body mass of adult males and adult females is 96 ± 2 and 201 ± 4 kg in spring, and 158 ± 4 and 273 ± 6 kg in autumn, respectively (Swenson *et al*. 2007). Thus, males are on average 1·7–1·8 times heavier than females. Annual cub mortality in the study area averages 35% (Swenson *et al*. 1997, 2001) and is highest during the mating season (Zedrosser *et al*. 2009). During an intensive field study (2008–2011), we confirmed that NPI caused cub loss in at least 92% of the detected events of cub loss (Steyaert 2012).

### Location data

We modelled resource selection based on locations from individual bears monitored with GPS (Global Positioning System) collars (GPS Plus; Vectronic Aerospace GmbH Berlin, Germany) during 2006–2010; see Arnemo *et al*. (2011) for details on capture and handling. The GPS collars delivered one position every 30 min, with an average fix rate of 94·2%. We removed GPS fixes with dilution of precision values ≥5 to increase spatial accuracy. This reduced the average fix success rate to 73·4%. We used the year a bear was monitored as the sample unit (bear-year = one bear followed for 1 year), and obtained data from the three reproductive classes. We defined the operational study area as the 95% kernel density estimated range of all GPS locations after data screening.

For every bear-year we sampled availability using random points, equal to the number of GPS points. We sampled availability in the operational study area, based on the principle that every individual could physically reach every site within this area [i.e. Manly's design type II (Manly *et al*. 2002)] and randomly assigned every data point to a training or validation data set with a 50% probability. We divided the data into the mating season (1 May–15 July) and the postmating season (1 August–1 October), with a 2-week break between (16 July–31 July) for a clear separation between them. We further divided the data into eight 3-hour intervals to cover diurnal variance in bear behaviour (1, 00:00–2:59; 2, 3:00–5:59; 3, 6:00–8:59; 4, 9:00–11:59; 5, 12:00–14:59; 6, 15:00–17:59; 7, 18:00–20:59; 8, 21:00–23:59).

### Spatial landscape data

We derived spatial landscape data from three sources, i.e. topographical map tiles (National Land Survey of Sweden, licence i 2012/901, http://www.lantmateriet.se), a digital elevation model (DEM, 50 × 50m pixel size, National Land Survey of Sweden, licence i 2012/901, http://www.lantmateriet.se) and Resourcesat1-IRS-P6-LISS3 satellite imagery (23·5 × 23·5m, imagery captured on 2 and 7 June 2007, available free at http://www.lantmateriet.se). We processed the satellite images with Erdas Imagine 9·1 (Leica Geosystems 2010), and used ArcGIS 9·2 (ESRI) to derive data from the DEM and topographical maps.

Human disturbance – Humans may have a profound impact on the distribution, population size and structure and behaviour of wildlife (Woodroffe, Thirgood & Rabinowitz 2005). We therefore selected human infrastructures, i.e. settlements (< 200 inhabitants), buildings (single standing buildings, such as cabins and hunting lodges), paved roads (termed ‘roads’), unpaved forest roads (termed ‘forest roads’) and trails from a topographical map. We derived the Euclidean distance to each of these for all 25 × 25 m pixels in the study area.

NDVI – We derived a Normalized Difference Vegetation Index (NDVI) map of the study area from the satellite imagery. The NDVI is a spectral vegetation index based on the reflectance of land-cover features of red and near-infrared electromagnetic energy, and is commonly used as a proxy for vegetation density (Pettorelli *et al*. 2005).

Terrain characteristics *–* We used the DEM to derive terrain ruggedness indices and slope steepness for each 50 × 50 m pixel in the study area. We calculated a terrain ruggedness index (TRI) for each cell based on the variation in its eight neighbouring cells in altitude, slope aspect, steepness and curvature (refer to Steyaert *et al*. 2012b for a detailed description). We categorized the TRI pixel values into four quartiles (class 1, least rugged, to 4, most rugged). Because behavioural responses to terrain ruggedness may vary with spatial scale (Mårell & Edenius 2006), we created a second terrain ruggedness index on the landscape scale (TRI1000). We calculated the average TRI for each pixel with a moving window, using all surrounding pixels within a 1000-m radius, and categorized the resulting map into the same four quartiles.

Water bodies *–* Water can affect the distribution of terrestrial wildlife (Main 2008). We derived the Euclidean distance to the closest creek (< 3 m wide) and larger water bodies for each 25 × 25 m pixel from the topographical maps.

Land cover – We obtained land-cover types through a supervised classification with a maximum likelihood classifier of the satellite imagery (87% overall user's accuracy) (Steyaert *et al*. 2012b). We considered the land-cover types ‘bog’, ‘young dense forest’, ‘young open forest’ and ‘older forest’ for further analysis. Other land-cover types were not considered for further analysis because of their near absence in the study area (e.g. ‘pasture’), or because of being unsuitable as bear habitat (e.g. ‘open water’, ‘human habitation’).

### Data analysis

We used logistic generalized linear mixed models with a logit link function and a Markov Chain Monte Carlo algorithm to model brown bear resource selection (Hadfield 2010). Models were run with 65 000 iterations, a burnin of 15 000, a thinning interval of 50 and an Inverse Whiskart prior. We used availability/use as the binary response variable, and a linear combination of the landscape variables as the explanatory variables. We included individual ‘bear ID’ and ‘year’ as random factors. We tested for collinearity among model variables with a Spearman Rho correlation test. We removed the variable ‘Slope’ from further analysis because it correlated highly (*ρ* = 0·606, *P* < 0·001) with TRI.

We formulated two candidate models *a priori* (Burnham & Anderson 2002), i.e. a global model including all variables (NDVI, bog, young open forest, young open forest, older forest, TRI, TRI1000 and distance to the nearest creek, water body, trail, forest road, road, building and settlement) and a reduced model containing only the variables we believed to be the strongest predictors of brown bear resource selection (NDVI, bog, young open forest, young open forest, older forest, TRI and distance to the nearest forest road and settlement). We selected the most parsimonious candidate model based on the Deviance Information Criteria (DIC) (Hadfield 2010). We used the potential scale reduction factor (PSRF) diagnostic to assess model convergence, based on the variance within and between duplicate Markov chains (Brooks & Gelman 1998). Model convergence is reached when PSRF values approach 1. We used the validation location data set to validate the predictive accuracy of the resource selection models (see Boyce *et al*. 2002 for a methodological description). We used the ‘MCMCglmm’ (Hadfield 2010) and the ‘coda’ package (Plummer *et al*. 2010) to model resource selection.

We created resource selection maps for each reproductive class, diurnal interval and season, based on the modelling results and the spatial data layers (Boyce *et al*. 2002). The pixel values of these maps indicate the relative probability that the pixel will be selected for by an individual of a given class during a given period of time (Boyce *et al*. 2002). The resource selection maps served as the basis for quantifying spatiotemporal segregation among the three classes. We extracted pixel values of spatially independent points from each map (refer to Hiemstra *et al*. 2009 for a theoretical and methodological description), and used Pearson product-moment correlation tests to quantify spatiotemporal segregation among the reproductive classes for each diurnal interval and season. Negative correlations in resource selection between reproductive classes suggest spatiotemporal segregation and avoidance, no correlation suggests spatiotemporal segregation and positive values indicate resource selection similarity. We used the ‘automap’ package (Hiemstra *et al*. 2009) in *R* to assess spatial autocorrelation in resource selection maps.

We evaluated the responses of the different reproductive classes towards the model variables to obtain insight in the mechanisms of spatiotemporal segregation. Therefore, we considered parameter estimates of a given ordinal or continuous variable as significant if its' 95% Highest Posterior Density (HPD) interval did not contain 0. We included land-cover classes in the regression models as nominal binary dummy variables. Because we were interested in the relative importance of each land-cover class in the brown bears' resource selection, we ranked the nominal land-cover classes of each model according to their parameter estimates (1, low – 4, high) and evaluated differences in the selection for land-cover types among reproductive classes and seasons with Friedman Rank Sum tests (Appendix S1). For all analyses, we considered α = 0·05 as the threshold level for statistical significance. We used R 2·12·0 for all statistical analyses (R Development Core Team 2009).

## Results

### Model evaluation

We modelled resource selection with data from 90 bear-years from 51 individuals, including 17 males (35 bear-years) and 34 females (55 bear-years), 17 of which had given birth at least once during the study period (21 bear-years). The operational study area encompassed 2,937 km^2^. We obtained 431,703 bear locations; the average number used in a training data set was 6,275 (range: 2,173–8,783; Appendix S2).

The global models performed better than all reduced models (Appendix S2), and were selected for further analyses. The PSRF approached 1 for each selected model (Appendix S2). Model validation showed that all resource selection functions had a good predictive accuracy (Appendix S2).

### Correlates in resource selection

Locations on resource selection maps became spatially independent on average at 3,443·6 m. We therefore added this distance to our sampling criteria for sampling random points to compare correlation in resource selection levels among reproductive classes. We extracted values from each resource selection map from 128 spatially independent random points. Examples of resource selection maps are presented in Appendix S3.

**Fig. 1:**
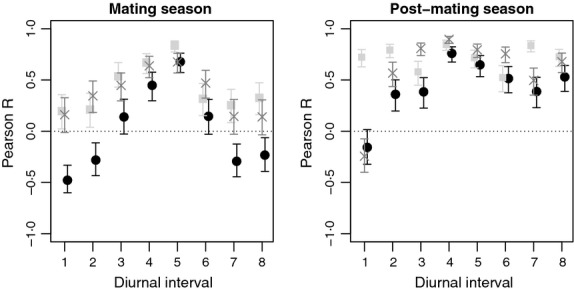
Correlation in resource selection among reproductive classes of brown bears. The Spearman correlation coefficients and their respective 95% confidence intervals are given for correlations in resource selection between adult males (≥5 years) and females with cubs-of-the-year (**•**), between lone adult females (≥5 years) and females with cubs-of-the-year (x) and between lone adult females and adult males (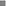) during eight 3-h intervals in the mating and postmating seasons in central Sweden during 2006–2010.

Resource selection correlations between adult males and lone females were always significantly positive and varied between 0·196 (*P* = 0·027, 00:00–2:59, mating season) and 0·846 (*P* < 0·001, 9:00–11:59, postmating season). During the mating season, correlations between the resource selection of females/cubs and adult males were significantly negative during night-time intervals (00:00–2:59, Pearson correlation coefficient (*r*) = −0·478, *P* < 0·001; 3:00–5:59, *r* = −0·281, *P* = 0·001; 18:00–20:59, *r* = −0·293, *P* < 0·001; 21:00–23:59, *r* = −0·234, *P* = 0·007) (Fig. 1). Positive correlations were found during the intervals from 9:00 to 11:59 (*r* = 0·447, *P* < 0·001) and 12:00 to 14:59 (*r* = 0·679. *P* < 0·001), and no correlations were evident between 6:00–8:59 (*r* = 0·140, *P* = 0·1) and 15:00–17:59 (*r* = 0·144, *P* = 0·105). After the mating season, resource selection correlations between females/cubs and adult males were always strongly and significantly positive (*P* < 0·001), with correlation coefficients between 0·147 and 0·759, except between 00:00 and 2:59, when no correlation was found (*r* = −0·158, *P* = 0·075) (Fig. 1). Resource selection correlations between females/cubs and lone females were mostly positive during both seasons, with the exception of the diurnal interval from 00:00 to 2:59 during the postmating season, when resource selection between lone females and females/cubs correlated negatively (*r* = −0·224, *P* = 0·005) (Fig. 1).

### Responses to landscape variables

To facilitate interpretation, we report and discuss our results based on graphical representations of the modelling results (Appendix S1 and S4). Parameter estimates of fixed effects, their 95% HPD intervals, and the MCMC-simulated *P*-values are available on request.

**Fig. 2:**
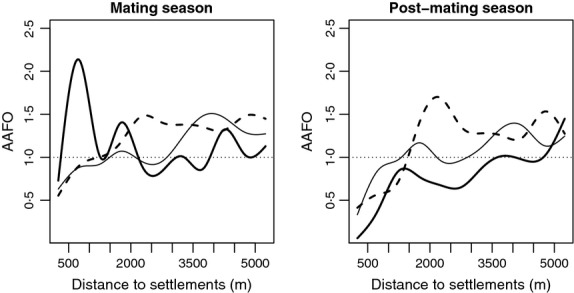
Area-adjusted frequency of occurrence (AAFO) of brown bears in relation to distance to settlements. AAFO values for adult males (≥5 years, —), adult lone female (≥5 years, **―**) and female brown bears with cubs-of-the-year (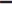) within a 5-km area (10 × 500m wide buffer zones) around settlements in the study area, fitted with spline smoothers, during the mating and postmating season in central Sweden during 2006–2010. Values >1 indicate that a given buffer area was used more relative to its availability.

Human disturbance – During the mating season, adult males selected areas further from buildings than random, there was no apparent selection by lone females, but females/cubs were generally closer to buildings than random (Appendix S4). After the mating season, all classes were farther from buildings than random (Appendix S4). During the mating season, adult males selected for areas closer to roads, forest roads and trails, especially during night (Appendix S4). Lone females avoided roads and selected areas closer to trails during night, but forest roads had no apparent effect (Appendix S4). Females/cubs generally avoided areas close to roads, forest roads and trails. During the postmating season, all reproductive classes were farther from forest roads and trails than random, especially during daytime (6:00–8:59, 9:00–11:59 and 12:00–14:59). Roads were generally avoided by adult males and lone females, whereas they had no apparent effect on females/cubs (Appendix S4). All reproductive classes generally selected for areas close to settlements during the mating season. During the postmating season, adult males selected areas near settlements, lone females showed no selection, but females/cubs avoided settlements (Appendix S4). Our finding that all categories selected areas closer to settlements during the mating season is counterintuitive (Woodroffe, Thirgood & Rabinowitz 2005). To examine this more closely, we plotted the area-adjusted frequency of occurrence (AAFO) of GPS positions for bears of all categories within a 5-km radius (divided into 500-m bands) around settlements (Fig. 2). AAFO values >1 indicate that an area unit is used more than expected. During the mating season, AAFO values for females/cubs exceeded 1 and peaked at 500–1000 m from settlements, but adult males avoided settlements closer than 1500 m (Fig. 2). For lone females, AAFO values fluctuated around 1, until approximately 3000 m from settlements, after which they exceeded 1. After the mating season, AAFO values for females/cubs exceeded 1 from distances around 3500 m from settlements and for lone females and adult males at approximately 1000 and 1500 m from settlements respectively (Fig. 2). Thus, during the mating season, females/cubs used areas close to settlements (500–1000 m) more than expected, whereas adult males and lone females avoided these areas. Based on the AAFO method, bears of all reproductive classes avoided settlements during the postmating season, and this effect was strongest for females/cubs.

**Fig. 3:**
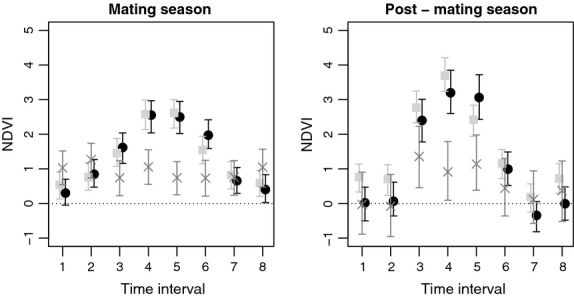
Diurnal and seasonal responses of brown bears to the Normalized Difference Vegetation Index (NDVI). Parameter estimates and their 95% highest posterior density intervals are shown for adult males (≥5 years, •), adult lone female (≥5 years, 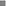) and females with cubs-of-the-year (x), during eight 3-h time intervals during the mating and postmating seasons in central Sweden during 2006–2010.

NDVI *–* Adult males and lone females showed a bell-shaped diurnal trend in their selection of areas with high NDVI values, peaking at midday during both seasons (Fig. 3). Females/cubs did not show this pattern during the mating season and parameter estimates for NDVI values were generally lower than for the other reproductive classes. The response to NDVI was similar among reproductive classes during the postmating season (Fig. 3).

Terrain characteristics – Local-scale selection for terrain ruggedness was similar for all reproductive classes during both seasons. All reproductive classes always selected for the most rugged terrain during the mating season (Appendix S4). During the postmating season, all reproductive classes selected for the most rugged terrain during nocturnal hours (Appendix S4). At the landscape scale during the mating season, adult males selected for the most rugged terrain, there was no apparent selection by lone females, but females/cubs showed a marked and consistent selection against rugged landscapes (Appendix S4). During the postmating season, all reproductive classes selected for the most rugged terrain (Appendix S4).

Water bodies *–* Creeks and larger water bodies generally did not affect resource selection by adult males and lone females during the mating season (Appendix S4), but females/cubs significantly avoided them during four of the eight diurnal intervals (Appendix S4). During the postmating season, creeks and larger water bodies were generally avoided by all reproductive classes (Appendix S4).

Land-cover types *–* Adult males and lone females did not select land-cover types uniformly during the mating season (adult males, Friedman χ^2^ = 16·35, d.f. = 3, *P* < 0·001; lone females, Friedman χ^2^ = 13·95, d.f. = 3, *P* = 0·002). Both classes then preferred young dense forest (see Appendix S1 for *post hoc* results). Females/cubs selected for young dense forest and older forest during the mating season (Friedman χ^2^ = 20·25, d.f. = 3, *P* < 0·001; Appendix S1). During the postmating season, adult males and lone females showed no clear preference for any land-cover type (adult males, Friedman χ^2^ = 3·15, d.f. = 3, *P* = 0·369, adult females, Friedman χ^2^ = 3, d.f. = 3, *P* = 0·391), whereas females/cubs then selected for young open and young dense forest (Friedman χ^2^ = 15·75, d.f. = 3, *P* = 0·001; Appendix S1).

## Discussion

Resource selection by brown bears in our study system varied seasonally, diurnally and among reproductive classes. Our results suggest that there was no apparent spatiotemporal segregation between adult males and lone females throughout the year (predictions H1a, H2a) and strong spatiotemporal segregation between adult males and females/cubs during most diurnal intervals during the mating season only (prediction H2b). We found some spatiotemporal segregation (3/8 diurnal intervals) between females/cubs and lone females during the mating season, whereas spatiotemporal segregation was not apparent during the postmating season (with the exception of one diurnal interval) (prediction H2c). Therefore, our results provide strongest support for the ‘NPI – sexual selection hypothesis’ (H2) to explain spatiotemporal segregation in our study system. We suggest that the ‘NPI – foraging/competition hypothesis’ (H1) plays a minor role in explaining spatiotemporal segregation because spatiotemporal segregation between adult males and females/cub or lone females and females/cubs was not apparent during the postmating season. We refute the ‘body size hypotheses’ (H3) for explaining spatiotemporal segregation in our study system because we did not observe clear spatiotemporal segregation between adult males and lone females throughout the year.

### Strategies and patterns in resource selection

We found that resource selection by the reproductive classes showed a diurnal and seasonal shift, probably due to changing physiology, food availability, human presence and infanticide risk. We suggest that resource selection of the three reproductive classes during the mating season reflects differences in the strength of sexual selection among the reproductive classes. Competition for mates is typically stronger among males than females, with males sometimes trading feeding for mate acquisition (Mysterud, Langvatn & Stenseth 2004). For example males of the extremely size-dimorphic northern elephant seal (*Mirounga angustirostris*) can lose up to one third of their body mass during the mating season (Deutsch, Haley & Le Boeuf 1990), and male moose (*Alces alces*) stop feeding during the rut (Mysterud, Solberg & Yoccoz 2005). Life-history theory predicts that females without dependent offspring optimize their body condition and fecundity by selecting areas with high-quality food resources and low predation risk (Partridge & Harvey 1988). We suggest that during the mating season, male brown bears invest more in mate acquisition than in feeding, lone females invest more in feeding and optimizing their body condition in comparison with males and females/cubs favour strategies to reduce the risk for NPI without compromising their own survival. After the mating season, we suggest that spatiotemporal segregation is less pronounced among the three reproductive classes because all are expected to optimize their body condition during hyperphagia prior to hibernation, and the three reproductive classes face a common risk, i.e. human hunting and related disturbances.

Spatiotemporal strategies to avoid NPI have been suggested in several species. Female Hanuman langurs and gorillas (*Gorilla gorilla*) with dependent young may disperse to avoid infanticide by males (Hrdy 1979). Female beluga whales (*Delphinapterus leucas*) with calves reside in open waters near the mainland, apparently to avoid interspecific predation and infanticidal males (Loseto *et al*. 2006). In carnivores, female lions with dependent offspring may avoid infanticidal males by becoming temporary nomads (Pusey & Packer 1994). Also in other brown bear populations, females with dependent offspring have been suggested to reduce infanticide risk by avoiding conspecifics (Wielgus & Bunnell 1994; Ben-David, Titus & Beier 2004). However, conclusive evidence for spatiotemporal strategies to avoid NPI is rare (Ebensperger & Blumstein 2007), and the adaptive significance of NPI often remains unexplained.

It is commonly accepted that animals can assess predation risk and behave accordingly. Animals may use landscape features or daylight as cues to evaluate risk (Brown & Kotler 2004; Creel & Christianson 2008). We suggest that females/cubs assess their environment for the occurrence of potentially infanticidal individuals, maybe by using food availability and human presence as cues. Females/cubs may avoid congregated food sources, such as salmon streams, salt marshes and garbage dumps, to avoid potentially infanticidal conspecifics (Craighead, Sumner & Mitchell 1995; Rode, Farley & Robbins 2006), resulting in a trade-off between nutrition and cub safety (Mattson & Reinhart 1995; Ben-David, Titus & Beier 2004). Pearson (1975), Wielgus & Bunnell (1994) and Swenson *et al*. (2001) suggested that females/cubs minimize infanticide risk by selecting the poorest habitats, such as alpine tundra and high-altitude rocky areas. Females/cubs have also been suggested to associate with humans to avoid aggressive males (Nevin & Gilbert 2005a; Rode, Farley & Robbins 2006).

We found that resource selection by different reproductive classes was a complex and multiscaled spatiotemporal mechanism, where females/cubs responded differently to landscape characteristics than the other classes, especially compared with adult males during the mating season. During this season, females/cubs selected the least rugged terrain on the landscape scale, avoided trails, forest roads and roads and selected areas relatively close to buildings and settlements. Also, they showed no diurnal trend in selection for patches with high NDVI values. Adult males, in contrast, selected the most rugged terrain on the landscape scale and areas close to all types of roads during the mating season. They avoided buildings and showed a strong diurnal pattern in selection for patches with high NDVI values. Except for selecting against areas close to roads during the mating season, resource selection of lone females was similar to that of adult males. However, the strength of selection coefficients for certain variables (e.g. terrain ruggedness on the local and the landscape scale) sometimes differed between adult males and lone females (Appendix S4).

Nocturnal behaviour in the brown bear is often suggested to result from human activity (Swenson 1999). In our study area, bear behaviour is closely linked to human disturbance (Martin *et al*. 2010; Ordiz *et al*. 2011) and bears show a period of low activity during daytime (9:00–18:00) (Moe *et al*. 2007). We found a diurnal component in the differential resource selection among the reproductive classes. Resource selection was most similar during daytime, suggesting that all bears perceive human disturbance as a threat then. Only females/cubs preferred older forests during night-time and crepuscular hours, when bear activity peaks (Moe *et al*. 2007) and human disturbance is low (Martin *et al*. 2010). We suggest that females/cubs perceive conspecifics as a greater threat than humans at these times and therefore select habitats that facilitate escape, such as older forest types, with more large trees that cubs can climb to escape potential perpetrators (Swenson 2003). Also, in older forest, females/cubs may detect potential perpetrators earlier by sight and possibly also by olfaction (Swenson 2003). In addition, adult males and lone females always avoided buildings and a zone of approximately 1500 m around settlements, whereas females/cubs strongly selected for areas close to buildings and settlements during the mating season. We suggest that females/cubs used areas close to humans (but not road infrastructure) as safety refuges. This is in accordance with McLellan & Shackleton (1988), Rode, Farley & Robbins (2006) and Nevin & Gilbert (2005b), who suggested that females with dependent offspring may associate with humans to avoid infanticide by males. The use of humans as a shield against predation has also been reported for North American ungulates (Berger 2007; Muhly *et al*. 2011).

### Alternative explanations

During spring and early summer, cub mobility may restrain maternal movement patterns (Dahle & Swenson 2003), possibly contributing to different resource selection between females/cubs and other reproductive classes. Some of our results support this; females/cubs selected the least rugged landscapes only during the mating season, when cubs are least mobile. The relation between terrain ruggedness and cub mobility is intuitive, but there is no plausible biological relationship between cub mobility and other significant model variables (e.g. NDVI). Dahle & Swenson (2003) attributed seasonal shifts in females/cubs' home range sizes to their secretive behaviour during the mating season, when the risk for SSI is high (Zedrosser *et al*. 2009), and less secretive behaviour after the mating season.

Martin *et al*. (2013) studied diurnal and seasonal movement patterns of female brown bears in relation to reproductive status (with/without cubs). They found that the probability of large-scaled daily displacement was higher for females/cubs than for lone females during the premating season and lower during the mating season. Martin *et al*. (2013) also showed that females/cubs can cover relatively large daily distances (with an average speed of 0·13 km h^−1^) during the mating season, but remain in a restricted area by having average turning angles close to 90°. Martin *et al*. (2013) suggest that female/cubs restrict their movements to reduce the risk of NPI.

Lactation is probably the most costly process in mammals, and can affect female movements and resource selection (Clutton-Brock, Albon & Guinness 1989). In bears, lactation lasts for 1·5–2·5 years, and peaks around August of the cubs' first year (Craighead, Sumner & Mitchell 1995; Farley & Robbins 1995). If lactation were a major factor affecting resource selection, we would expect similar patterns of resource selection by females/cubs during both the mating season and the postmating season. Therefore, we suggest that physiological aspects of lactation did not strongly affect the resource selection by females/cubs.

### Safety comes with costs

Perceived predation risk alters the prey's behaviour (Brown, Kotler & Bouskila 2001), irrespective of whether predation is inter- or intraspecific (Nevin & Gilbert 2005b). These behaviour-mediated effects can affect prey population fitness through restricted resource selection, induced habitat change, elevated stress, etc. (Brown, Kotler & Bouskila 2001; Creel *et al*. 2005). In the brown bear, one effect of NPI risk is reduced consumption of high-quality foods (Mattson & Reinhart 1995; Ben-David, Titus & Beier 2004; Rode, Farley & Robbins 2006) and subsequently reduced production of cubs (Wielgus & Bunnell 2000) and population growth (Wielgus *et al*. 2001). Mattson & Reinhart (1995) showed that females consuming cutthroat trout (*Oncorhynchus clarki*) at spawning streams were less fecund than females that avoided spawning streams, and explained this by high intraspecific predation risk where bears aggregated. In addition, competition for resources adds to the female cost of reproduction (Stearns 1989). Infanticide can thus also increase the ecological costs for females through behaviour-mediated effects (Mattson & Reinhart 1995).

## Conclusion

We found that resource selection is a complex mechanism, varying spatiotemporally and among reproductive classes. We suggest that differential resource selection among reproductive classes was a consequence of sex-specific reproductive strategies, with females/cubs adapting their resource selection during the mating season to avoid potentially infanticidal males, probably using human presence as a safety refuge. We found little or no support for two other hypotheses to explain spatiotemporal segregation, ‘NPI – foraging/competition hypothesis’ and the ‘body size hypothesis’. After the mating season, when SSI cannot any longer be beneficial as a male reproductive strategy, the resource selection of adult males, lone females and females/cubs becomes similar. We suggest that bears then adapt a strategy to build up fat reserves prior to hibernation and adapt their resource selection to cope with a common risk factor, i.e. the human hunter. We suggest that individuals use landscape cues to assess their environment and the risk factors therein and select their resources accordingly. In species exhibiting SSI, female avoidance of infanticidal males may be more common than reported because proximate and ultimate causes of sex-specific behavioural strategies are difficult to disentangle (Ebensperger & Blumstein 2007; Singh *et al*. 2010). Therefore, we suggest that fitness effects of infanticide avoidance behaviour be studied to provide ultimate explanations. Given that the rate of infanticide can depend on the rate of hunting of males (Swenson *et al*. 1997; Zedrosser *et al*. 2009) and that infanticide risk can affect resource selection and mediate other behaviours, we suggest that more research be directed towards the cascade that may flow from hunting, through infanticide and towards female fitness and their reproductive costs.

## Supporting Information

**Appendix S1.** Rank scores and post hoc comparisons of bear responses towards land-cover types.

**Appendix S2.** Model diagnostics of the resource selection models.

**Appendix S3.** Examples of resource selection maps.

**Appendix S4.** Graphical representations of the modelling results.
