# Supplementary material for: Male reproductive strategy explains spatiotemporal segregation in brown bears
Source: J Anim Ecol. 2013 Mar 5;82(4):836–45. doi: 10.1111/1365-2656.12055 (PMC3757318; doi:10.1111/1365-2656.12055)
Supplement: Supplementary file 1 [file jane0082-0836-SD1.doc]

Rank scores and post hoc comparisons of bear responses towards land cover types.

1. Rank scores (1-4) for the land cover types ‘Bog’, ‘Young open forest’, ‘Young dense forest’, and ‘Older forest’ for adult male, lone female, and female brown bears with cubs-of-the-year during 8 3-hour diurnal intervals in the mating and postmating seasons in central Sweden from 2006-2010. μ and σ denote the mean and the standard error for each land cover type per season and reproductive class, respectively. Gray cells identify the response of a habitat type that significantly (α = 0.05) contributed to resource selection at a given diurnal interval for a given reproductive class.

|  | Diurnal interval: | 00:00 - 2:59 | 3:00 - 5:59 | 6:00 - 8:59 | 9:00 - 11:59 | 12:00 - 14:59 | 15:00 - 17:59 | 18:00 - 20:59 | 21:00 - 23:59 | μ | σ |
| --- | --- | --- | --- | --- | --- | --- | --- | --- | --- | --- | --- |
| males, mating season | Bog | 3 | 1 | 1 | 1 | 1 | 1 | 2 | 2 | 1.5 | 0.76 |
|  | Young open forest | 2 | 3 | 2 | 2 | 2 | 2 | 3 | 3 | 2.38 | 0.52 |
|  | Young dense forest | 4 | 4 | 4 | 4 | 4 | 4 | 4 | 4 | 4 | 0 |
|  | Older forest | 1 | 2 | 3 | 3 | 3 | 3 | 1 | 1 | 2.13 | 0.99 |
| females/cubs, mating season | Bog | 1 | 1 | 1 | 1 | 1 | 1 | 1 | 1 | 1 | 0 |
|  | Young open forest | 2 | 2 | 2 | 2 | 2 | 3 | 2 | 2 | 2.13 | 0.35 |
|  | Young dense forest | 3 | 3 | 4 | 4 | 4 | 4 | 4 | 3 | 3.63 | 0.52 |
|  | Older forest | 4 | 4 | 3 | 3 | 3 | 2 | 3 | 4 | 3.25 | 0.71 |
| lone females, mating season | Bog | 3 | 2 | 1 | 1 | 1 | 1 | 2 | 2 | 1.63 | 0.74 |
|  | Young open forest | 1 | 3 | 2 | 2 | 2 | 4 | 3 | 3 | 2.5 | 0.93 |
|  | Young dense forest | 4 | 4 | 4 | 4 | 4 | 3 | 4 | 4 | 3.88 | 0.35 |
|  | Older forest | 2 | 1 | 3 | 3 | 3 | 2 | 1 | 1 | 2 | 0.93 |
| males, post-mating season | Bog | 4 | 3 | 1 | 1 | 1 | 2 | 3 | 4 | 2.38 | 1.3 |
|  | Young open forest | 2 | 2 | 3 | 2 | 3 | 3 | 2 | 3 | 2.5 | 0.53 |
|  | Young dense forest | 3 | 4 | 4 | 4 | 4 | 4 | 1 | 1 | 3.13 | 1.36 |
|  | Older forest | 1 | 1 | 2 | 3 | 2 | 1 | 4 | 2 | 2 | 1.07 |
| females/cubs, post-mating season | Bog | 2 | 1 | 1 | 1 | 1 | 1 | 1 | 1 | 1.13 | 0.35 |
|  | Young open forest | 3 | 4 | 3 | 3 | 3 | 3 | 4 | 4 | 3.38 | 0.52 |
|  | Young dense forest | 1 | 3 | 4 | 4 | 4 | 4 | 3 | 3 | 3.25 | 1.04 |
|  | Older forest | 4 | 2 | 2 | 2 | 2 | 2 | 2 | 2 | 2.25 | 0.71 |
| lone females, post-mating season | Bog | 4 | 1 | 1 | 1 | 1 | 1 | 3 | 4 | 2 | 1.41 |
|  | Young open forest | 2 | 3 | 3 | 2 | 3 | 3 | 4 | 2 | 2.75 | 0.71 |
|  | Young dense forest | 1 | 4 | 4 | 4 | 4 | 4 | 2 | 1 | 3 | 1.41 |
|  | Older forest | 3 | 2 | 2 | 3 | 2 | 2 | 1 | 3 | 2.25 | 0.71 |

1. Mean rank scores for land cover types (B = bog, YO = young open forest, YD = young dense forest, O = older forest) per season and brown bear reproductive class (adult males (white bars), lone females (black bars), and females with cubs-of-the-year (gray bars)) in central Sweden from 2006-2010. The whiskers represent the mean + 1 standard error for a given class/land cover type. Characters (a, b, and ab) indicate significant (α = 0.05) differences between mean scores.


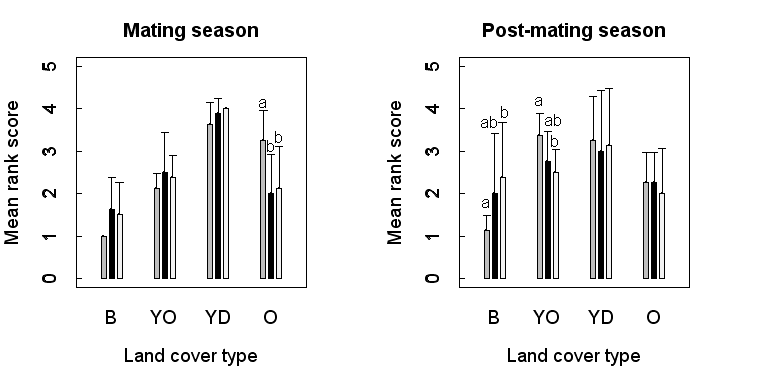


1. Post-hoc test results

Adult males: young dense forest always received the highest score (4) during all diurnal intervals, and was ranked significantly higher than young open forest (μ = 2.38, σ = 0.52, p < 0.001), older forest (μ = 2.13, σ = 0.99, p < 0.001), and bogs (μ = 1.5, σ = 0.76, p < 0.001).

Lone females: young dense forest was the highest ranked land cover type (μ = 3.88, σ = 0.35) during the mating season, and ranked higher than the other land cover types (young open forest: μ = 2.50, σ = 0.93, p = 0.005; older forest: μ = 2.0, σ = 0.93, p < 0.001, bogs: μ = 1.63, σ = 0.74, p < 0.001.

Females with cubs-of-the-year: We found no significant differences between rank scores of young dense forest (with the highest rank μ = 3.63, σ = 0.52) and older forest (μ = 3.25, σ = 0.71, p = 0.442), but both were ranked higher than young open forest (μ = 2.13, σ = 0.25, p_older forest_ = 0.005, p_young dense forest_ = 0.001), and bogs (μ = 1.00, σ = 0.00, p_older forest_ < 0.001, p_young dense forest_ < 0.001). During the postmating season, young open forest received the highest score (μ = 3.38, σ = 0.52), but was not significantly different from young dense forest, which was ranked as the second highest land cover type (μ = 3.25, σ = 0.52, p = 0.999). Young open forest was then preferred above older forest, but not above young dense forest (μ = 2.25, σ = 0.71, p_young open forest_ = 0.013, p_young dense forest_ = 0.074). Bogs were always the least preferred land cover class by females/cubs during the postmating season (μ = 1.13, σ = 0.35, p_all classes_ < 0.001).
