# Supplementary material for: Male reproductive strategy explains spatiotemporal segregation in brown bears
Source: J Anim Ecol. 2013 Mar 5;82(4):836–45. doi: 10.1111/1365-2656.12055 (PMC3757318; doi:10.1111/1365-2656.12055)
Supplement: Supplementary file 2 [file jane0082-0836-SD2.doc]

Model diagnostics of brown bear resource selection models for adult males (≥ 5 years), lone adult females (≥ 5 years), and females with cubs-of-the-year during the mating and the postmating seasons in central Sweden (2006-2010). The global model was always selected as the most parsimonious. The ‘*psrf’* indicates the Gelman diagnostic for model convergence, *N* = sample size for each specific model, ‘ts’ indicates the diurnal interval, *ρ* = the Spearman Rho correlation coefficient between the area-adjusted frequency of bear positions (validation dataset) and the binned resource selection probability class (training dataset) for each model, and *p* = the p value of the Spearman Rho correlation between the area adjusted frequency of bear positions and the binned resource selection probability class for each model. ∆DIC indicates the difference in DIC values between the two candidate models.

|  | **Mating season** | | | | | | | **Post-mating season** | | | |
| --- | --- | --- | --- | --- | --- | --- | --- | --- | --- | --- | --- |
|  | *ts* | *∆DIC* | *psrf* | *N* | *ρ* | *p* | *∆DIC* | *psrf* | *N* | *ρ* | *p* |
| **Adult males** | 00:00 - 2:59 | -55.75 | 1.02 | 7378 | 0.97 | < 0.001 | -119.24 | 1.02 | 5547 | 0.999 | < 0.001 |
|  | 3:00 - 5:59 | -49.31 | 1.02 | 8477 | 0.999 | < 0.001 | -220.77 | 1.01 | 6927 | 0.929 | 0.002 |
|  | 6:00 - 8:59 | -51.28 | 1.01 | 7252 | 0.999 | < 0.001 | -83.32 | 1.01 | 4748 | 0.999 | < 0.001 |
|  | 9:00 - 11:59 | -30.1 | 1.02 | 7052 | 0.999 | < 0.001 | -129.28 | 1.02 | 4870 | 0.999 | < 0.001 |
|  | 12:00 - 14:59 | -34.35 | 1.02 | 7262 | 0.881 | < 0.001 | -122.5 | 1.01 | 4952 | 0.964 | 0.003 |
|  | 15:00 - 17:59 | -39.36 | 1.02 | 7613 | 0.994 | < 0.001 | -109.51 | 1.02 | 6011 | 0.905 | 0.005 |
|  | 18:00 - 20:59 | -62.51 | 1.01 | 8507 | 0.922 | 0.001 | -175.36 | 1.01 | 6423 | 0.976 | < 0.001 |
|  | 21:00 - 23:59 | -58.36 | 1.01 | 6912 | 0.999 | < 0.001 | -79.58 | 1.01 | 4988 | 0.994 | < 0.001 |
| **Lone females** | 00:00 - 2:59 | -80.31 | 1.01 | 8540 | 0.976 | < 0.001 | -134 | 1.02 | 7382 | 0.976 | < 0.001 |
|  | 3:00 - 5:59 | -95.88 | 1.01 | 8561 | 0.976 | < 0.001 | -223.14 | 1.02 | 8417 | 0.999 | < 0.001 |
|  | 6:00 - 8:59 | -96.17 | 1.01 | 8496 | 0.999 | < 0.001 | -248.87 | 1.02 | 8071 | 0.999 | < 0.001 |
|  | 9:00 - 11:59 | -128.78 | 1.02 | 8507 | 0.999 | < 0.001 | -226.77 | 1.02 | 8138 | 0.952 | 0.001 |
|  | 12:00 - 14:59 | -109.95 | 1.03 | 8488 | 0.999 | < 0.001 | -237.67 | 1.02 | 8195 | 0.999 | < 0.001 |
|  | 15:00 - 17:59 | -113.21 | 1.01 | 8615 | 0.976 | < 0.001 | -263.43 | 1.01 | 8163 | 0.976 | < 0.001 |
|  | 18:00 - 20:59 | -111.38 | 1.01 | 8783 | 0.905 | 0.005 | -143.07 | 1.02 | 8242 | 0.999 | < 0.001 |
|  | 21:00 - 23:59 | -107.19 | 1.01 | 8430 | 0.976 | < 0.001 | -124.12 | 1.01 | 7508 | 0.999 | < 0.001 |
| **Females/cubs** | 00:00 - 2:59 | -92.17 | 1.02 | 5373 | 0.999 | < 0.001 | -86.27 | 1.02 | 2222 | 0.964 | 0.003 |
|  | 3:00 - 5:59 | -103.48 | 1.01 | 5515 | 0.994 | < 0.001 | -142.81 | 1.02 | 2325 | 0.999 | < 0.001 |
|  | 6:00 - 8:59 | -116.14 | 1.02 | 5634 | 0.976 | < 0.001 | -128.26 | 1.02 | 2405 | 0.97 | < 0.001 |
|  | 9:00 - 11:59 | -77.01 | 1.01 | 5746 | 0.97 | < 0.001 | -179.97 | 1.02 | 2554 | 0.994 | < 0.001 |
|  | 12:00 - 14:59 | -75.3 | 1.01 | 5666 | 0.999 | < 0.001 | -172.12 | 1.02 | 2670 | 0.952 | 0.001 |
|  | 15:00 - 17:59 | -69.15 | 1.01 | 5615 | 0.994 | < 0.001 | -121.88 | 1.02 | 2357 | 0.893 | 0.012 |
|  | 18:00 - 20:59 | -85.19 | 1.01 | 5695 | 0.999 | < 0.001 | -131.39 | 1.01 | 2362 | 0.999 | < 0.001 |
|  | 21:00 - 23:59 | -80.31 | 1.02 | 5434 | 0.994 | < 0.001 | -116.36 | 1.01 | 2173 | 0.994 | < 0.001 |
