# Supplementary material for: Male reproductive strategy explains spatiotemporal segregation in brown bears
Source: J Anim Ecol. 2013 Mar 5;82(4):836–45. doi: 10.1111/1365-2656.12055 (PMC3757318; doi:10.1111/1365-2656.12055)

Details of resource selection maps of brown bears; adult males (A), females with cubs-of-the-year (B) and lone adult females (C) during the mating season and the postmating season (D, E, F, respectively) between 03:00 and 5:59. Water bodies are marked blue, light tones indicate high relative probabilities of use.


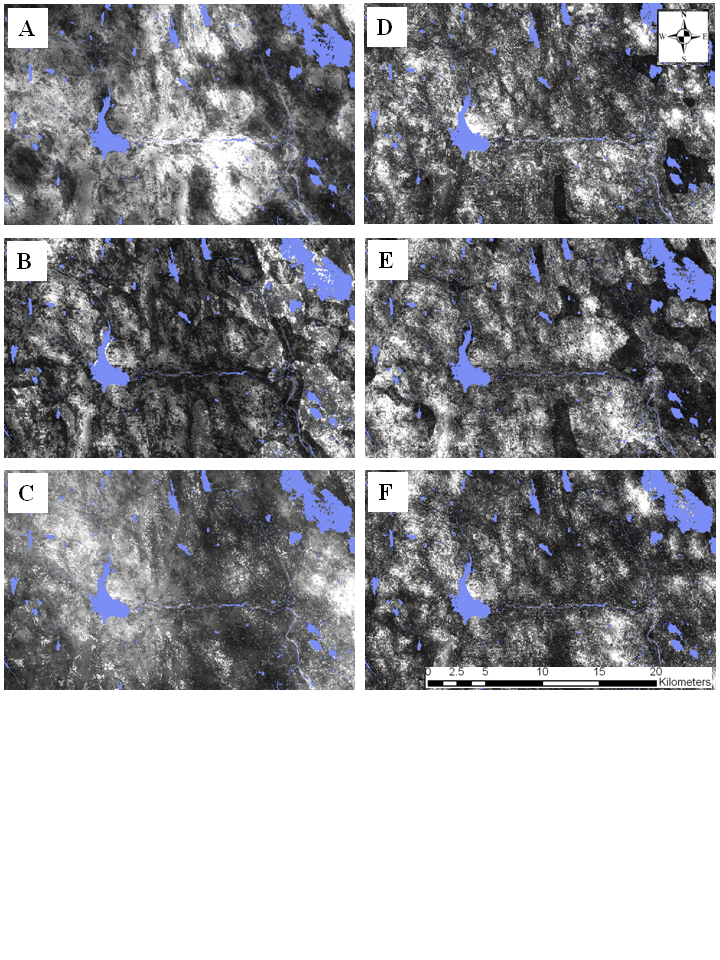

Supplement: Supplementary file 3 [file jane0082-0836-SD3.doc]
