# Supplementary material for: Male reproductive strategy explains spatiotemporal segregation in brown bears
Source: J Anim Ecol. 2013 Mar 5;82(4):836–45. doi: 10.1111/1365-2656.12055 (PMC3757318; doi:10.1111/1365-2656.12055)

Graphical representations of the modeling results. Parameter estimates and their 95% highest posterior density intervals of the continuous and ordinal categorical model variables for adult male (≥ 5 years, ●), adult lone female (≥ 5 years, ■) and female brown bears with cubs-of-the-year (x), during 8 3-hour time intervals during the mating and postmating seasons in central Sweden during 2006-2010. Parameter estimates, 95% Highest Posterior Density intervals, and MCMC-simulated p-values for each variable in for each of the 48 model are available on request.

**Human Disturbance**


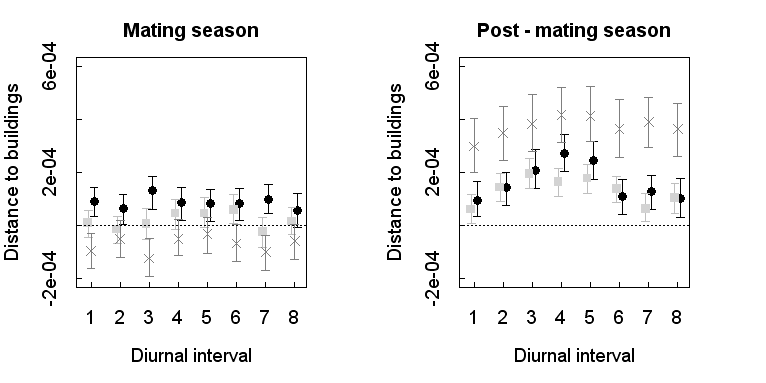

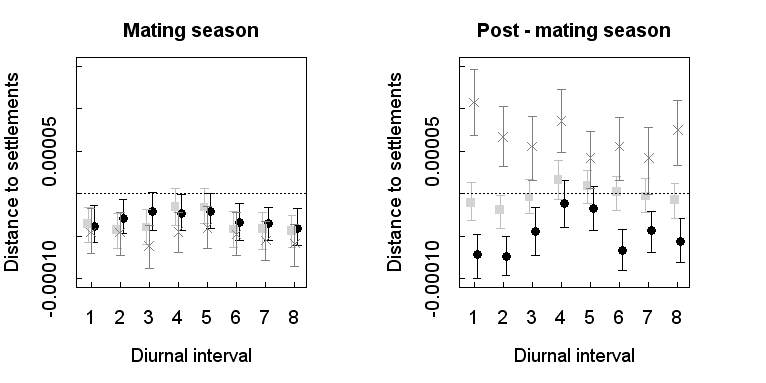


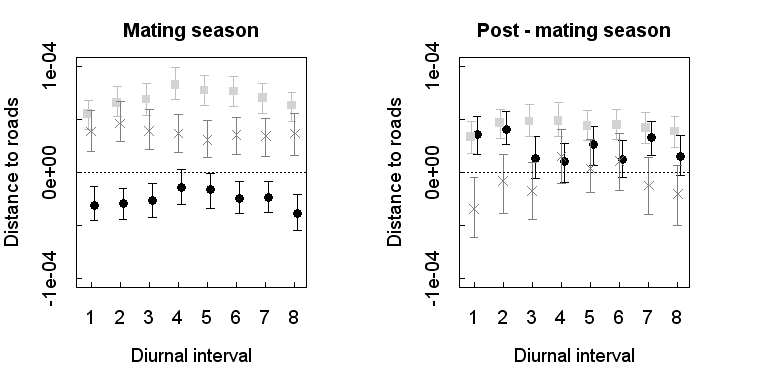


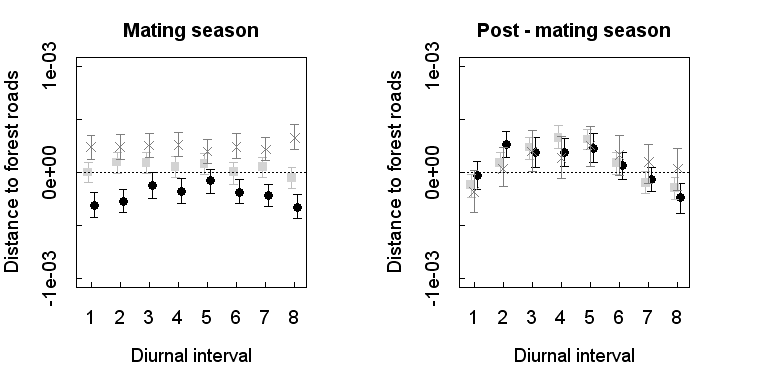


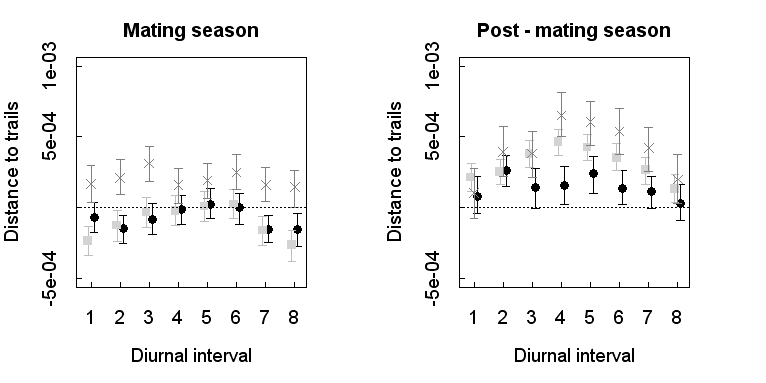


**Terrain characteristics**


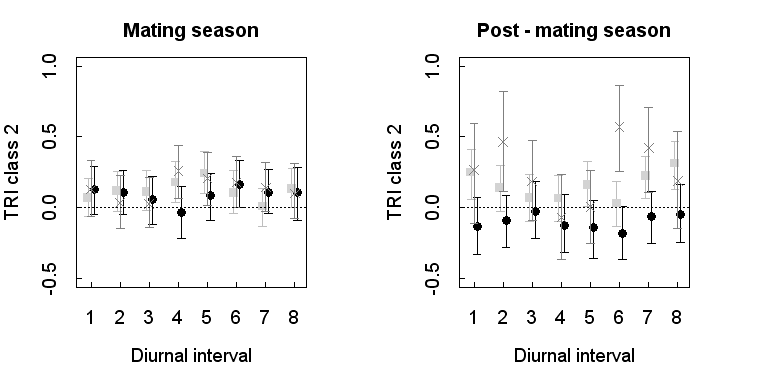

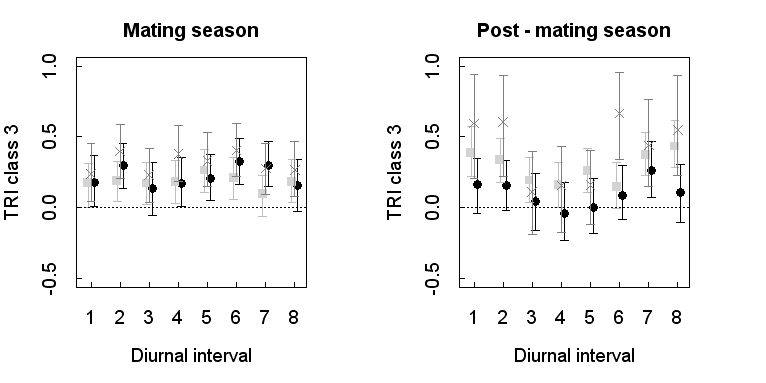

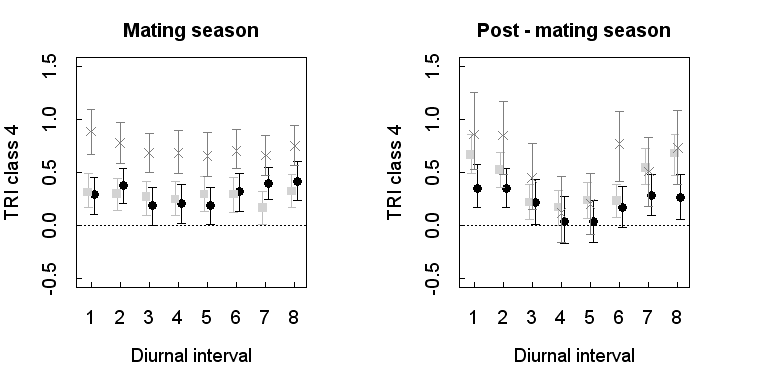


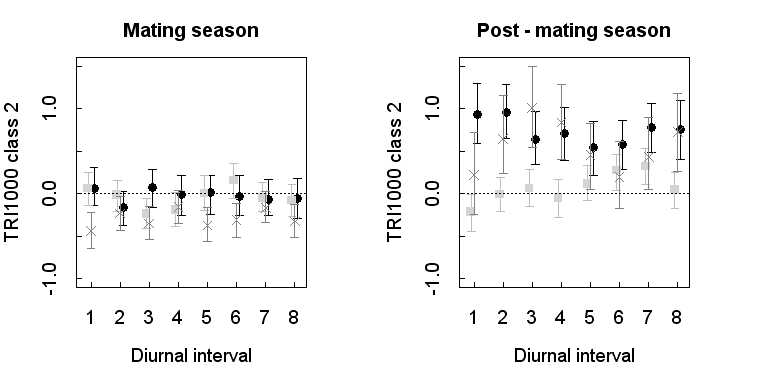

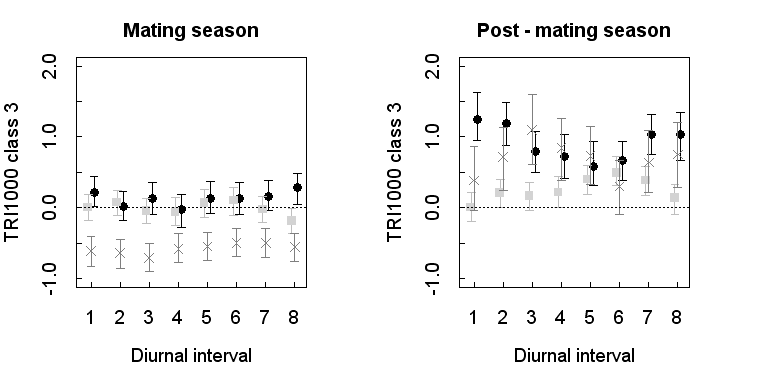

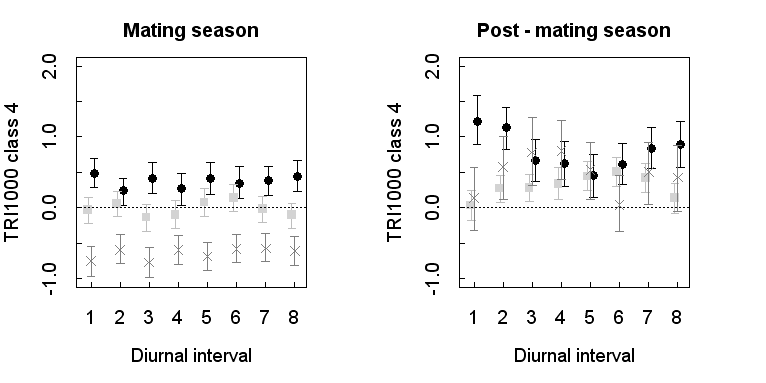


**Water bodies**


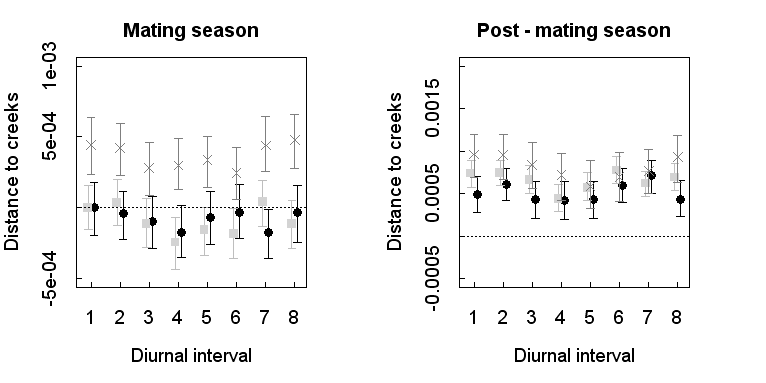


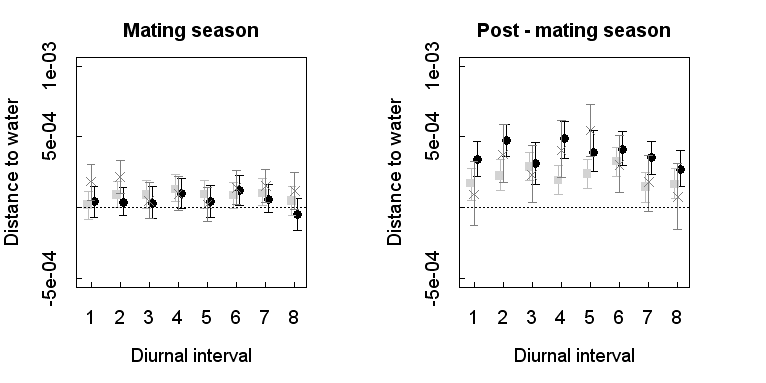

Supplement: Supplementary file 4 [file jane0082-0836-SD4.doc]
